# Glasgow‐Blatchford score combined with nasogastric aspirate as a new diagnostic algorithm for patients with nonvariceal upper gastrointestinal bleeding

**DOI:** 10.1002/deo2.185

**Published:** 2022-11-14

**Authors:** Toshiyuki Wakatsuki, Tomohiko Mannami, Shinichi Furutachi, Hiroki Numoto, Tsuyoshi Umekawa, Mayu Mitsumune, Tsukasa Sakaki, Hanako Nagahara, Yasushi Fukumoto, Takashi Yorifuji, Shin'ichi Shimizu

**Affiliations:** ^1^ Department of Gastroenterology National Hospital Organization Okayama Medical Center Okayama Japan; ^2^ Department of Epidemiology, Okayama University Graduate School of Medicine Dentistry and Pharmaceutical Sciences Okayama Japan

**Keywords:** endoscopy, gastrointestinal hemorrhage, nasogastric intubation, peptic ulcer, risk assessment

## Abstract

**Objectives:**

The Glasgow‐Blatchford score (GBS) is a widely used risk assessment tool for patients with upper gastrointestinal bleeding. However, it only identifies a relatively low proportion of patients at low risk for adverse events and poor outcomes. We developed a simple diagnostic algorithm combining the GBS and nasogastric aspirate and evaluated its diagnostic performance.

**Methods:**

A total of 115 consecutive patients with suspected nonvariceal upper gastrointestinal bleeding who underwent nasogastric tube placement and upper endoscopy at our emergency department were prospectively evaluated. We compared the diagnostic accuracy of the GBS and our algorithm for predicting high‐risk endoscopic lesions (HRELs) using receiver operating characteristic curve analysis.

**Results:**

Thirty‐five patients had HRELs. Compared with the GBS, our algorithm showed superior performance with respect to the prediction of HRELs (area under the curve, 0.639 and 0.854, respectively; *p* < 0.001). With set optimal threshold values, the algorithm identified a significantly higher proportion of patients who did not have HRELs than the GBS (23.5% vs. 2.6%, *p* < 0.001).

**Conclusions:**

The novel algorithm has improved the diagnostic performance of the GBS and predicted more patients who did not have HRELs than the GBS alone. After further validation, it may be a useful tool for making clinical management decisions for patients with nonvariceal upper gastrointestinal bleeding.

## INTRODUCTION

Endoscopy is important in the diagnosis and treatment of patients with upper gastrointestinal bleeding (UGIB). According to international clinical guidelines, emergent endoscopy and endoscopic hemostatic therapy for patients with high‐risk endoscopic lesions (HRELs) improve prognostic results.^1^ Nevertheless, many patients do not require emergent endoscopy. Up to 80% of patients who undergo endoscopy for suspected UGIB do not have HRELs.^2^, ^3^, ^4^, ^5^, ^6^ Therefore, predicting the need for endoscopic treatment would aid emergency department (ED) physicians in making prompt and appropriate clinical decisions and allocating healthcare resources optimally by avoiding emergent endoscopy when it is dispensable.

Several risk stratification tools have been developed to predict the outcomes of UGIB patients.^7^, ^8^, ^9^, ^10^, ^11^, ^12^, ^13^ The most widely used and best studied is the Glasgow‐Blatchford score (GBS), which is superior in predicting the need for hospital‐based intervention or death.^7^, ^14^, ^15^ The GBS also helps identify UGIB patients at very low risk for adverse events and poor outcomes. Patients with a GBS of zero can avoid hospital admission and be managed as outpatients.^3^, ^16^, ^17^, ^18^ However, it identifies a relatively low proportion of low‐risk patients (8%–16% of those suspected of having UGIB).^3^, ^15^

Nasogastric tube placement and aspirate evaluation have also been used to stratify risk in patients with suspected UGIB. This diagnostic bedside procedure has the advantages of availability, low cost, and very low risk of complications.^19^, ^20^ Bloody nasogastric aspirate (NGA) is associated with active bleeding, HRELs detected by endoscopy, and increased incidence of recurrent bleeding.^21^, ^22^, ^23^, ^24^ However, routine nasogastric tube placement for suspected acute UGIB is controversial because the negative predictive value of NGA is low and tube insertion can cause discomfort in patients.^21^, ^24^, ^25^ To date, few studies have included NGA appearance as a factor in a risk stratification model.^26^, ^27^, ^28^

In this study, we developed a novel prediction algorithm that combines GBS and NGA and evaluated its diagnostic performance to determine whether this algorithm would demonstrate better discriminative ability in predicting the presence of HRELs and whether it would identify more patients without HRELs than the GBS alone.

## METHODS

### Patients and study design

This was a single‐center, prospective, observational study conducted at the National Hospital Organization Okayama Medical Center. Of consecutive adult patients with suspected UGIB who visited our ED, those who underwent nasogastric tube placement and subsequent upper endoscopy were enrolled. The decision to perform nasogastric tube placement and/or emergent endoscopy was at the discretion of the attending physician in the ED and the consulted gastroenterologist. We defined patients with suspected UGIB as those who presented with hematemesis, coffee‐ground vomiting, and melena. Patients with impaired consciousness or suspected esophageal/gastric varices were excluded. We excluded patients with suspected esophageal/gastric varices because some clinicians consider this a contraindication to nasogastric tube insertion, although no published clinical trials suggest that nasogastric lavage worsens variceal bleeding.^29^ Clinical and laboratory findings were recorded, and the GBS was calculated according to the original criteria (Table S1).^7^ Endoscopic examination was performed within 12 h after the presentation. This study was approved by the Institutional Review Board at Okayama Medical Center (2018‐154) and was conducted in accordance with the Declaration of Helsinki. We obtained informed consent from all patients before enrolment. Since this was an observational study of usual care and involved no randomization or intervention, enrolment in a public trial registry was not performed.

### Nasogastric tube placement and aspirate appearance

Nasogastric tube placement and aspirate evaluation were performed by the initial attending doctors in the ED. Aspirate characteristics were recorded as bloody, coffee‐ground, bile‐like, or clear. Aspirates containing bright red or dark red blood were classified as bloody NGAs; those matching the latter three descriptions were classified as non‐bloody NGAs. Typical images of NGAs are shown in Table 1. If nothing was aspirated by syringe, 50 ml of saline was carefully injected, and then aspiration was repeated.^29^

**TABLE 1:** Sample images of nasogastric aspirate findings

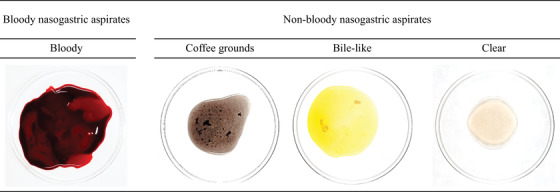

### Upper endoscopy and other treatment procedures

Upper endoscopy was performed by a board‐certified endoscopist who documented the cause of bleeding, evaluated the need for endoscopic treatment, and developed the treatment plan. The treatment outcomes for each patient, including surgery or interventional radiology procedures, were documented. Information on 30‐day mortality due to UGIB was collected.

### Definition of HRELs

The primary outcome of this study was HRELs, which were defined as peptic ulcers with class Ia (spurting/gushing bleeding), Ib (oozing bleeding), or IIa (non‐bleeding with visible blood vessel) lesions, according to the Forrest classification.^30^ These are the endoscopic findings that justify endoscopic intervention, as per international consensus statements.^1^ When the bleeding source was non‐peptic ulcer related, HRELs were defined to be spurting/gushing bleeding.

### Algorithm combining GBS and NGA

We developed an algorithm that combined GBS and NGA results to stratify patients with suspected nonvariceal UGIB (NVUGIB) into two groups. One group comprised patients with non‐bloody NGA and GBS ≤a certain threshold, considered low‐risk. The other group consisted of the remaining patients (patients with bloody NGA or GBS > the threshold, considered high‐risk). We subsequently estimated and compared the diagnostic accuracy of the GBS with our newly devised algorithm.

### Statistical analysis

Continuous variables presented as mean and standard deviation were compared using a Student's two‐sample t‐test. Categorical variables expressed as numbers (*n*) and percentages (%) were compared using the chi‐square and Fisher's exact tests. Discriminative analysis of the GBS and newly devised algorithm for predicting the presence of HRELs was performed by comparing the area under the curve (AUC) for the receiver operating characteristic (ROC), using the Delong model.^31^ For this comparison, the algorithm was described with continuous variables as follows: if NGA was bloody, 23 points were added to the GBS; however, the total score was limited to 23 points. This was consistent with our algorithm, which was designed to predict the presence of HRELs whenever the NGA was bloody. Continuous net reclassification improvement (NRI) and integrated discrimination improvement (IDI) were calculated to assess the added predictive ability of the algorithm to the GBS.^32^ The optimal score thresholds to exclude patients with HRELs were identified based on sensitivity ≥98%. This value is based on previous prospective studies with relatively large sample sizes, in which the sensitivity of a score threshold to identify low‐risk patients was 98%–100%.^4^, ^15^, ^17^ We emphasized sensitivity because reduced sensitivity with the use of a higher threshold value would lead to an increased risk of misclassifying patients requiring treatment. The diagnostic performances of the GBS and algorithm were assessed by calculating the sensitivity, specificity, positive predictive value, and negative predictive value with 95% confidence intervals (CIs). Statistical significance was set at *p* < 0.05. No sample size calculations were conducted before the study. However, the number of enrolled patients with and without HRELs provided post‐hoc power of 0.84 to detect differences in the AUC for the ROC between the two models with a two‐sided significance level of *p* < 0.05 for predicting HRELs. All statistical analyses were performed using R version 3.6.1 (The R Foundation for Statistical Computing, Vienna, Austria).

## RESULTS

### Patient characteristics

A flow diagram of patient selection is shown in Figure S1. A total of 115 patients were enrolled in this study between December 2018 and October 2021. The patients’ baseline characteristics are presented in Table 2. On endoscopic examination, 35 patients (30.4%) had HRELs and were endoscopically treated. The mean time from the visit to the upper endoscopy was 191 ± 129 min. There were no differences in patients’ characteristics, laboratory data, or medication between the groups of patients with and without HRELs. The most common endoscopic diagnosis in both groups was a gastric ulcer, which was more common in patients with HRELs.

**TABLE 2:** Patient characteristics and outcomes

		High‐risk endoscopic lesions		
	Total (*n* = 115)	With (*n* = 35)	Without (*n* = 80)	*p*‐value^†^
Age, mean ± SD, years	74.8 ± 18.3	74.4 ± 11.0	74.9 ± 16.0	0.86
Male	73 (63.5)	24 (68.6)	49 (61.3)	0.531
Symptoms				
Hematemesis	66 (57.4)	19 (54.3)	47 (58.8)	0.686
Black stool	68 (59.1)	25 (71.4)	43 (53.8)	0.099
Syncope	6 (5.2)	3 (8.6)	3 (3.8)	0.367
Clinical features				
Systolic blood pressure, mean ± SD, mmHg	117.0 ± 23.8	112.54 ± 26.8	119.0 ± 22.2	0.182
Diastolic blood pressure, mean ± SD, mmHg	66.1 ± 17.8	62.3 ± 18.3	67.8 ± 17.4	0.127
Pulse, mean ± SD, bpm	90.4 ± 18.3	90.3 ± 20.7	91.3 ± 20.5	0.160
Hemoglobin, mean ± SD, mg/dl	8.21 ± 2.83	7.92 ± 2.29	8.33 ± 3.04	0.479
Blood urea nitrogen, mean ± SD, mg/dl	41.0 ± 29.5	43.8 ± 24.0	39.8 ± 31.7	0.499
Medical history				
Hepatic disease	9 (7.8)	4 (11.4)	5 (6.2)	0.452
Cardiac failure	7 (6.1)	4 (11.4)	3 (3.8)	0.197
Renal failure	9 (7.8)	3 (8.6)	6 (7.5)	1.000
Drug use				
Antiplatelet agent	34 (29.6)	8 (22.9)	26 (32.5)	0.377
Anticoagulant drug	20 (17.4)	6 (17.1)	14 (17.5)	1.000
Warfarin	8 (7.0)	2 (5.7)	6 (7.5)	1.000
DOAC	12 (10.4)	4 (11.4)	8 (10.0)	1.000
Steroid	7 (6.1)	3 (8.6)	4 (5.0)	0.433
PPI	36 (31.3)	12 (34.3)	24 (30.0)	0.667
Endoscopic findings				
Esophageal ulcer	3 (2.6)	1 (2.9)	2 (2.5)	1.000
Esophageal cancer	2 (1.7)	0 (0)	2 (2.5)	1.000
Mallory–Weiss syndrome	8 (7.0)	1 (2.9)	7 (8.8)	0.432
Gastric ulcer	40 (34.8)	22 (62.9)	18 (22.5)	<0.001
Gastric angioectasia	10 (8.7)	4 (11.4)	6 (7.5)	0.490
Gastric cancer	6 (5.2)	1 (2.9)	5 (6.3)	0.666
Duodenal ulcer	12 (10.4)	6 (17.1)	6 (7.5)	0.182
Treatment				
Endoscopic intervention	35 (30.4)	35 (100)	0 (0)	<0.001
Surgery	0 (0)	0 (0)	0 (0)	1.000
Interventional radiology	0 (0)	0 (0)	0 (0)	1.000
Mortality	0 (0)	0 (0)	0 (0)	1.000
GBS, mean ± SD, points**^‡^**	10.0 ± 4.2	11.3 ± 3.7	9.4 ± 4.2	0.020
Nasogastric aspirate findings				
Bloody	35 (30.4)	24 (68.6)	11 (13.8)	<0.001
Non‐bloody	80 (69.6)	11 (31.4)	69 (86.2)	
Coffee grounds	52 (45.2)	8 (22.9)	44 (55.0)	
Bile‐like	6 (5.2)	0 (0)	6 (7.5)	
Clear	22 (19.1)	3 (8.6)	19 (23.8)	

Data are *n* (%) unless otherwise defined.

^†^ Comparisons for continuous and categorical variables are made using Student's two‐sample t‐test and Fisher's exact test respectively.

^‡^ GBS can range from 0 to 23, with higher scores indicating higher risk.

Abbreviations: DOAC, direct oral anticoagulants; GBS, Glasgow‐Blatchford score; PPI, proton pump inhibitor; SD, standard deviation.

There was no enrolled patient for whom varices were considered the source of bleeding. Of the non‐enrolled 151 cases, five (4.3%) patients had variceal bleeding, all of whom were excluded for suspected esophageal/gastric varices (*n* = 16). However, there was no significant difference between enrolled and non‐enrolled patients in the percentage of variceal bleeding (*p* = 0.08). None of the patients underwent surgery or interventional radiology or died.

### GBS and NGA

The GBS value was significantly higher in patients with HRELs than in those without HRELs (11.3 ± 3.7 vs. 9.4 ± 4.2, *p* = 0.02) (Table 2). Regarding the NGA, 35 (30.4%) patients showed a bloody appearance and 80 (69.6%) had a non‐bloody appearance (Table 2). In all cases, NGA evaluation was feasible. Bloody aspirate was more common in patients with HRELs (68.6% vs. 13.8%, *p* < 0.001). The sensitivity, specificity, positive predictive value, and negative predictive value for predicting the presence of HRELs by bloody aspirate were 68.6%, 86.2%, 68.6%, and 86.2%, respectively.

**FIGURE 1:**
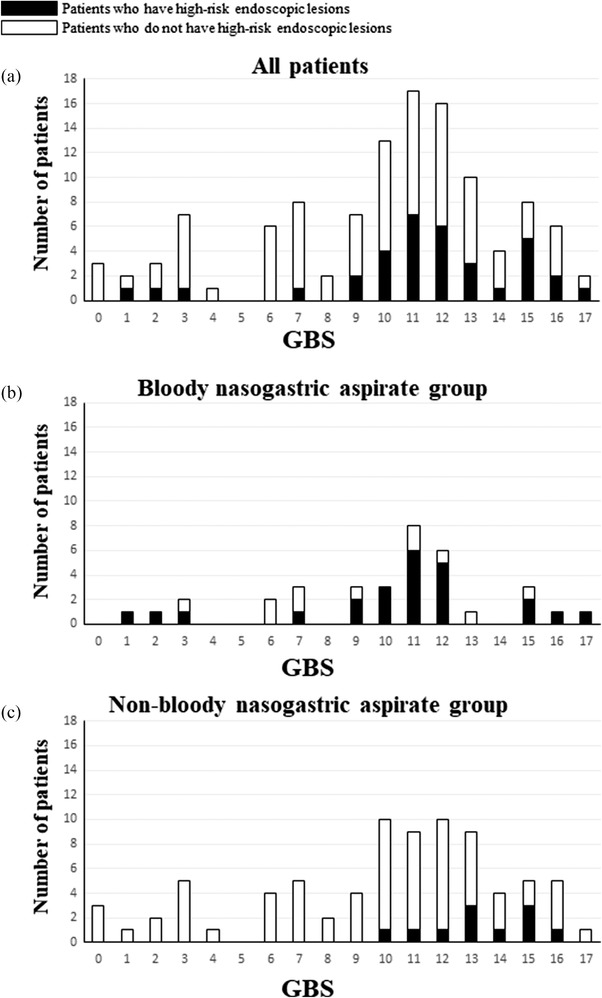
Distribution of patients with and without high‐risk endoscopic lesions according to their GBS values (a), in a group where the nasogastric aspirate had a bloody appearance (b), and in a group where the nasogastric aspirate had a non‐bloody appearance (c). The black bars represent patients with high‐risk endoscopic lesions, whereas the white bars represent those without high‐risk endoscopic lesions. GBS, Glasgow–Blatchford score.

A histogram plot showed that a higher GBS was associated with HREL presence (Figure 1a–c). In the histogram that included all patients (Figure 1a), none of the three patients‐ with GBS ≤0 had HRELs (2.6%). Of the 35 patients in the bloody NGA group (Figure 1b), 24 (68.6%) had HRELs, whereas 11 (31.4%) did not. These 11 patients represent false positive results in predicting HREL presence using bloody NGA. Endoscopic findings of these cases and their respective GBS values are shown in Table S2. Of the 80 patients in the non‐bloody NGA group (Figure 1c), 69 (86.2%) did not have HRELs, whereas 11 (13.8%) had HRELs. Endoscopic findings for these patients, representing false negative results, are shown in Table S3. No HRELs were identified in individuals with GBS scores ≤9 in the non‐bloody NGA group.

### Comparison of diagnostic accuracy between GBS and the algorithm

**FIGURE 2:**
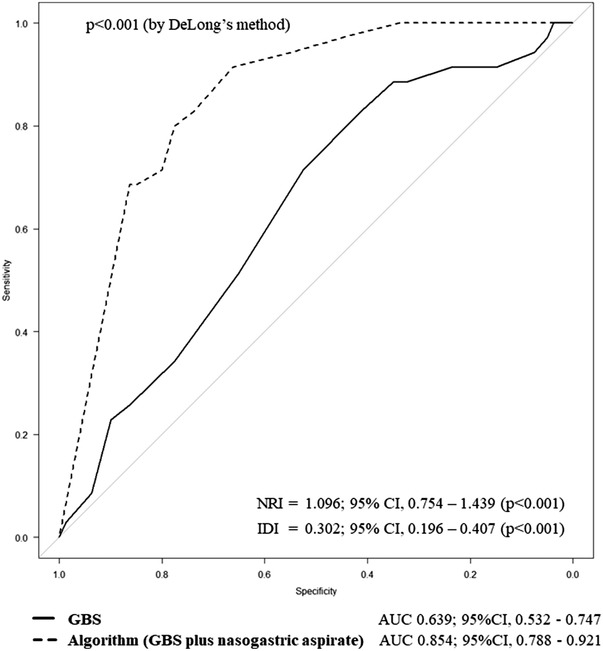
Comparison of AUC values between the GBS and our algorithm combining the GBS and nasogastric aspirate for the prediction of the presence of high‐risk endoscopic lesions. AUC, area under the curve; CI, confidence interval; GBS, Glasgow‐Blatchford score; IDI, integrated discrimination improvement; NRI, net reclassification improvement.

The AUC was 0.639 (95% CI, 0.532–0.747) for the GBS and 0.854 (95% CI, 0.788–0.921) for our algorithm (Figure 2). The results of the ROC analysis showed that our algorithm was superior to the GBS in predicting HREL presence (*p* < 0.001). Continuous NRI and IDI also demonstrated that the algorithm had a discriminative ability superior to the GBS (Figure 2).

Table 3 shows the sensitivity and specificity of the GBS and the algorithm at each threshold of the incorporated GBS values to exclude patients with HRELs. The optimal score thresholds for the GBS and algorithm were ≤0 and ≤9, respectively.

**TABLE 3:** Sensitivity and specificity of Glasgow–Blatchford score and our algorithm at each threshold to rule out patients who have high‐risk endoscopic lesions

GBS					Algorithm (GBS plus nasogastric aspirate)^†^				
Cut‐off	Patients who have high‐risk endoscopic lesions, *n* (%)	Patients who do not have high‐risk endoscopic lesions, *n* (%)	Sensitivity (%)	Specificity (%)	Cut‐off^‡^	Patients who have high‐risk endoscopic lesions, *n* (%)	Patients who do not have high‐risk endoscopic lesions, *n* (%)	Sensitivity (%)	Specificity (%)
0	0 (0)	3 (2.6)	100	3.8	0	0 (0)	3 (2.6)	100	3.8
≤1	1 (0.9)	4 (3.5)	97.1	5.0	≤1	0 (0)	4 (3.5)	100	5.0
≤2	2 (1.7)	6 (5.2)	94.3	7.5	2≤2	0 (0)	6 (5.2)	100	7.5
3≤3	3 (2.6)	12 (10.4)	91.4	15.0	3≤3	0 (0)	11 (9.6)	100	13.8
4≤4	3 (2.6)	13 (11.3)	91.4	16.2	≤4	0 (0)	12 (10.4)	100	15.0
≤5	3 (2.6)	13 (11.3)	91.4	16.2	≤5	0 (0)	12 (10.4)	100	15.0
≤6	3 (2.6)	19 (16.5)	91.4	23.8	≤6	0 (0)	16 (13.9)	100	20.0
≤7	4 (3.5)	26 (22.6)	88.6	32.5	≤7	0 (0)	21 (18.3)	100	26.2
≤8	4 (3.5)	28 (24.3)	88.6	35.0	≤8	0 (0)	23 (20.0)	100	28.8
≤9	6 (5.2)	33 (28.7)	82.9	41.2	≤9	0 (0)	27 (23.5)	100	33.8
≤10	10 (8.7)	42 (36.5)	71.4	52.5	≤10	1 (0.9)	36 (31.3)	97.1	45.0
≤11	17 (14.8)	52 (45.2)	51.4	65.0	≤11	2 (1.7)	44 (38.3)	94.3	55.0
≤12	23 (20.0)	62 (53.9)	34.3	77.5	≤12	3 (2.6)	53 (46.1)	91.4	66.2
≤13	26 (22.6)	69 (60.0)	25.7	86.2	≤13	6 (5.2)	59 (51.3)	82.9	73.8
≤14	27(23.5)	72 (62.6)	22.9	90.0	≤14	7 (6.1)	62 (53.9)	80.0	77.5
≤15	32 (27.8)	75 (65.2)	8.6	93.8	≤15	10 (8.7)	64 (55.7)	71.4	80.0
≤16	34 (29.6)	79 (68.7)	2.9	98.8	≤16	11 (9.6)	68 (59.1)	68.6	85.0
≤17	35 (30.4)	80 (69.6)	0	100	≤17	11 (9.6)	69 (60.0)	68.6	86.2

^†^ The algorithm to stratify suspected UGIB patients into two groups is as follows. One group comprises patients with non‐bloody nasogastric aspirate and a GBS value equal to or below a certain cut‐off level; another group comprises the rest of the patient, that is, those with bloody nasogastric aspirate or a GBS value more than a certain cut‐off level.

^‡^ Cut‐off value of GBS which is incorporated into the algorithm.

Abbreviations: GBS, Glasgow‐Blatchford score; UGIB, upper gastrointestinal bleeding.

**FIGURE 3:**
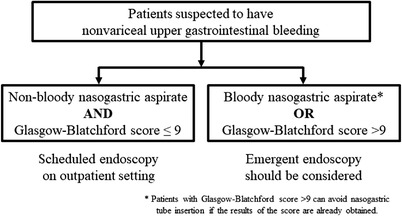
Algorithm combining the Glasgow‐Blatchford score and nasogastric aspirate appearance to evaluate the need for emergent endoscopy in patients presenting with suspected nonvariceal upper gastrointestinal bleeding.

The performance characteristics of the GBS and our algorithm when the above‐mentioned threshold values were set are listed in Table 4. Our algorithm identified a significantly higher proportion of patients who did not have HRELs than the GBS (23.5% vs. 2.6%, *p* < 0.001). The 27 patients classified as having no HRELs by the algorithm corresponded to those with GBS ≤9 in the histogram of the non‐bloody NGA group (Figure 2c). The remaining patients who were not classified as such by the algorithm corresponded to those with GBS > 9 in the histogram of the non‐bloody NGA group (Figure 2c) or those with any GBS in the histogram of the bloody NGA group (Figure 2b). Figure 3 shows the proposed management algorithm for patients with suspected NVUGIB to evaluate the necessity of emergent endoscopy.

**TABLE 4:** Performance characteristics of the Glasgow‐Blatchford score and our novel algorithm

	**Cut‐off**	**Patients not to have high‐risk endoscopic lesions** **^†^** **, (*n*) (%)**	**Sensitivity % (95% CI)**	**Specificity % (95% CI)**	**PPV % (95% CI)**	**NPV % (95% CI)**
GBS	≦0	3 (2.6)	100 (85.5–100)	3.8 (0.8–10.6)	100 (19.4–100)	31.2 (22.8–40.7)
Algorithm (GBS plus nasogastric aspirate)	≦9^‡^	27 (23.5)	100 (85.5–100)	33.8 (23.6–45.2)	100 (81.7–100)	39.8 (29.5–50.8)

^†^ A Comparison is made using the Chi‐square test. The algorithm identified a significantly higher proportion of patients who did not have high‐risk endoscopic lesions than GBS (*p* < 0.001).

^‡^ Cut‐off value of GBS which is incorporated into the algorithm.

Abbreviations: CI, confidence interval; GBS, Glasgow‐Blatchford score; NPV, negative predictive value; PPV, positive predictive value.

## DISCUSSION

This is the first prospective observational study to evaluate the efficacy of a risk stratification system that combines GBS and NGA in patients with suspected UGIB. Our algorithm showed better discriminative ability in predicting HREL presence than the GBS alone. Additionally, the algorithm excluded patients with HRELs better than the GBS alone, thus increasing the proportion of patients predicted to not require endoscopic treatment.

Of the 242 patients remaining after the exclusion of those who met the exclusion criteria, many did not undergo endoscopy and/or nasogastric tube placement. However, 68.6% (166/242) underwent endoscopy, a comparable rate to previous prospective findings of 68.9%–79.8%, where the clinical utility of GBS was evaluated.^4^, ^14^, ^15^ Similarly, 55.0% (133/242) of patients underwent nasogastric tube placement, also comparable to previous findings of 52.6%–67.3%, where the clinical utility of NGA was evaluated.^21^, ^22^ Therefore, we consider that the enrolled patients in this study are well representative of the NVUGIB population.

Few published studies have evaluated the efficacy of incorporating NGA into risk stratification systems. Adamopoulos et al. prospectively investigated UGIB patients and showed that patients with and without HRELs on endoscopy could be differentiated using an integer‐based scoring system that used four clinical and laboratory variables including NGA.^26^ By calculating NRI and IDI, another study showed that the addition of NGA to the GBS improved performance in predicting the presence of UGIB over GBS alone.^28^ The results of these studies support the suitability of incorporating NGA into a risk stratification system for UGIB, although our results are inconsistent with another retrospective study, where authors argued that NGA added limited benefit to the GBS when NGA appearance was scored and added to the GBS.^27^ However, there were some problems with their study. For example, the coffee‐ground appearance, which is less likely to indicate severe UGIB,^24^, ^29^ was also interpreted as a positive NGA, and only one point is assigned for a positive NGA. We believe that including NGA with an appropriate study setting and proper weighting increases the discriminative ability of the GBS.

The second important clinical observation in our study is that the algorithm better‐excluded patients with HRELs than the GBS alone. Several studies have proposed to extend the GBS threshold from 0 to 1 or more to increase the proportion of predicted lower‐risk patients.^4^, ^14^, ^15^, ^33^, ^34^ A recent large, prospective, multicenter, observational study involving 3012 UGIB patients showed that a score of ≤1 could be used to identify a low‐risk cohort, with the proportion increasing from 8.6% for GBS = 0 to 19.2% for GBS ≤1.^15^ Some studies also demonstrate that this threshold can be safely extended to 2.^14^, ^34^ Another approach is to modify the GBS by setting different thresholds or scoring the GBS differently according to age. However, an increase of only up to 2.3‐fold in the proportion of low‐risk patients predicted was reported, with limited benefit at an increased rate.^4^, ^5^, ^14^ Our algorithm, which incorporates NGA into the GBS, identified a remarkably higher proportion of patients without HRELs, with an approximately 10‐fold increase (2.6% vs. 23.5%) compared with the GBS alone. When considering our study in the context of extending the GBS threshold, our results showed that by incorporating NGA into an algorithm, we could extend the GBS threshold from 0 to as high as 9 without compromising safety.

The strength of our algorithm is that it is simple and consists of only two widely accepted existing indicators. It can be easily used by non‐gastroenterologists without the need to relearn an entirely new predictive model and will allow wider use of outpatient and scheduled endoscopy management. Although it is recommended that UGIB patients undergo endoscopy within 24 h after the presentation, not all facilities can afford the healthcare services proposed by international clinical guidelines. The results of nationwide audits in the UK have shown that only 50%–65% of patients underwent endoscopy within 24 h at night or on weekends.^35^, ^36^ The situation is less favorable in many economically resource‐poor countries, where endoscopy is unaffordable for most UGIB patients.^37^ Our simple algorithm could potentially contribute to the clinical practice of NVUGIB in a variety of settings in both developed and developing countries where endoscopists, support staff, or equipment needed for endoscopy are limited.

### Limitations

First, this study was conducted at a single center and considered data from only Japanese patients. Therefore, our results may not apply to other populations. Second, the sample size was relatively small. With a larger sample size, a certain number of patients with HRELs might appear in the low‐risk population with lower GBS and non‐bloody NGA, thereby reducing the diagnostic performance of our algorithm through a decline in sensitivity. Third, about half of the consecutive patients with suspected UGIB were not enrolled in the calculations used to establish our algorithm, which may have led to selection bias. Fourth, an algorithmic approach was employed in this study for simplicity of use. Therefore, it is unclear whether the combination of GBS and NGA is beneficial when using the approach of developing a scoring model based on a logistic regression model, which is the standard method for creating a prediction model. Finally, we were unable to conduct internal or external validation studies. External validation is necessary to determine a prediction model's reproducibility and generalizability to new and different patients. However, according to a questionnaire survey of 17 facilities belonging to the National Hospital Organization Gastroenterology Research Group, conducted as a preliminary survey for this study, the rate of patients experiencing HRELs in emergent endoscopies performed in 2016 was 31.9% (730/2291) (unpublished data). The similarity between this percentage and ours, 30.4% of HRELs, suggests that background factors are not markedly different, and the results of our study are potentially relevant to UGIB clinical practice in secondary and tertiary care centers in Japan. Further studies in a prospective, multicenter setting would clarify the usefulness of this algorithm and whether the GBS cutoff of 9 points is acceptable.

## CONCLUSION

A novel algorithm combining the GBS and NGA improved the diagnostic performance of the GBS and predicted more patients without HRELs than the GBS alone. More studies are encouraged to verify whether this algorithm would contribute to the clinical practice of treating NVUGIB, as a risk stratification system for guiding clinical management decisions.

## CONFLICT OF INTEREST

None.

## Supporting information


**Figure S1** Flow diagram of patients enrolled in this study. NGT, nasogastric tube

**Table S1** Glasgow‐Blatchford score
**Table S2** Endoscopic findings and respective Glasgow‐Blatchford scores in patients with bloody nasogastric aspirate despite having no high‐risk endoscopic lesions
**Table S3** Endoscopic findings in patients for whom nasogastric aspirate appearance was bloody versus non‐bloody
