# Supplementary figures and images for: Glasgow‐Blatchford score combined with nasogastric aspirate as a new diagnostic algorithm for patients with nonvariceal upper gastrointestinal bleeding
Source: DEN Open. 2022 Nov 14;3(1):e185. doi: 10.1002/deo2.185 (PMC9663679; doi:10.1002/deo2.185)

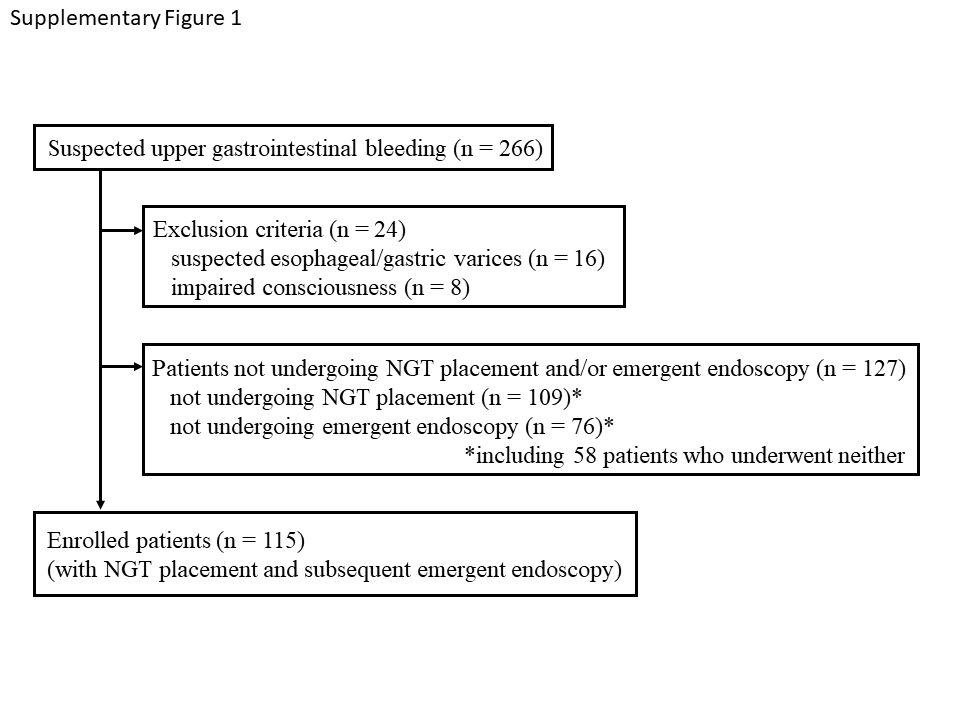

Supplement: Supplementary file 1 — Figure S1 Flow diagram of patients enrolled in this study. NGT, nasogastric tube [file DEO2-3-e185-s002.tif]
